# Microbead-Encapsulated Luminescent Bioreporter Screening of *P. aeruginosa* via Its Secreted Quorum-Sensing Molecules

**DOI:** 10.3390/bios14080383

**Published:** 2024-08-08

**Authors:** Abraham Abbey Paul, Yael Schlichter Kadosh, Ariel Kushmaro, Robert S. Marks

**Affiliations:** 1 Avram and Stella Goldstein-Goren Department of Biotechnology Engineering, Ben-Gurion University of the Negev, Be’er Sheva 84105, Israel; paulab@post.bgu.ac.il (A.A.P.); shlicter@post.bgu.ac.il (Y.S.K.); arielkus@bgu.ac.il (A.K.); 2 The Ilse Katz Center for Nanoscale Science and Technology, Ben-Gurion University of the Negev, Be’er Sheva 84105, Israel; 3 School of Sustainability and Climate Change, Ben-Gurion University of the Negev, Be’er Sheva 84105, Israel

**Keywords:** alginate, microbeads, optical biosensors, bioencapsulation, autoinducers, *Pseudomonas aeruginosa*, quorum sensing, poly-lysine, hydrogels, C_4_-HSL, 3-oxo-C_12_-HSL, Furanone C-30, QS inhibitor, whole-cell biosensors

## Abstract

*Pseudomonas aeruginosa* is an opportunistic Gram-negative bacterium that remains a prevalent clinical and environmental challenge. Quorum-sensing (QS) molecules are effective biomarkers in pinpointing the presence of *P. aeruginosa*. This study aimed to develop a convenient-to-use, whole-cell biosensor using *P. aeruginosa* reporters individually encapsulated within alginate-poly-L-lysine (alginate-PLL) microbeads to specifically detect the presence of bacterial autoinducers. The PLL-reinforced microbeads were prepared using a two-step method involving ionic cross-linking and subsequent coating with thin layers of PLL. The alginate-PLL beads showed good stability in the presence of a known cation scavenger (sodium citrate), which typically limits the widespread applications of calcium alginate. In media containing synthetic autoinducers—such as N-(3-oxo dodecanoyl) homoserine lactone (3-oxo-C_12_-HSL) and *N*-butanoyl-L-homoserine lactone (C_4_-HSL), or the cell-free supernatants of planktonic or the flow-cell biofilm effluent of wild *P. aeruginosa* (PAO1)—the encapsulated bacteria enabled a dose-dependent detection of the presence of these QS molecules. The prepared bioreporter beads remained stable during prolonged storage at 4 and −80 °C and were ready for on-the-spot sensing without the need for recovery. The proof-of-concept, optical fiber-based, and whole-cell biosensor developed here demonstrates the practicality of the encapsulated bioreporter for bacterial detection based on specific QS molecules.

## 1. Introduction

*Pseudomonas aeruginosa* is an increasingly prevalent opportunistic Gram-negative bacterium that causes nosocomial and life-threatening infections of immunocompromised [1] and cystic fibrotic patients [2]. Many Gram-negative bacteria regulate the expression of specialized gene sets in response to their population density by autoinduction, in which small molecules called autoinducers (AIs) are produced and can diffuse freely across the bacterial cell wall [3]. In response to the levels of AIs, individual cells can determine whether the population density is high enough to initiate the expression of a particular phenotype. This type of gene regulation is termed quorum sensing (QS) [3].

The study of QS has emerged as a key research area in the past two decades due to its involvement in vital microbial activities. For instance, bacterial swarming and motility, sporulation, biofilm formation, bioluminescence, and the production of virulence factors [4] are regulated by QS. Moreover, about 80% of microbial infections have been associated with biofilm formation, and the contribution of QS molecules in biofouling is well established. *N*-acyl homoserine lactones (AHLs) are used by *P. aeruginosa* as autoinducers for quorum sensing. The length of the fatty acyl group in the AHLs varies from 4 to 14 carbons, and the group can be either fully reduced, have a 3-oxo or 3-hydroxyl group, or have an unsaturated bond. *P. aeruginosa* signal molecules–*N*-butyryl-homoserine lactone (C_4_-HSL) and *N*-3-oxo-dodecanoyl-homoserine lactone (3OC_12_-HSL) have been detected in clinical samples of cystic fibrosis patients [5,6,7,8], with positive correlations found with the infection rate [9].

A highly sensitive detection of AHLs can be achieved by biosensors and liquid and gas chromatographic methods [10,11]. Although such physical–chemical techniques can be sensitive, their widespread use is hampered by the need for solvent extraction, derivatization of the analytes, and the cost of the instrument, as well as the requirement for trained personnel. Consequently, biosensors have shown to be viable alternatives due to their affordable cost, fast analysis time, and high sensitivity, thus making them ideal for routine testing and the screening of samples [10]. The bioreporter bacteria strain can detect quorum-sensing molecules because they are genetically rendered incapable of producing AHLs themselves but maintaining their capability of sensing their presence. Such genetic mutations are usually introduced upstream of a LuxR-controlled promoter, and they are fused to a gene coding for an easily detectable output signal. For details on the construction of bacterial *lux*-biosensors, readers are directed to the review work of Bazhenov et al. [12].

**Figure 1:**
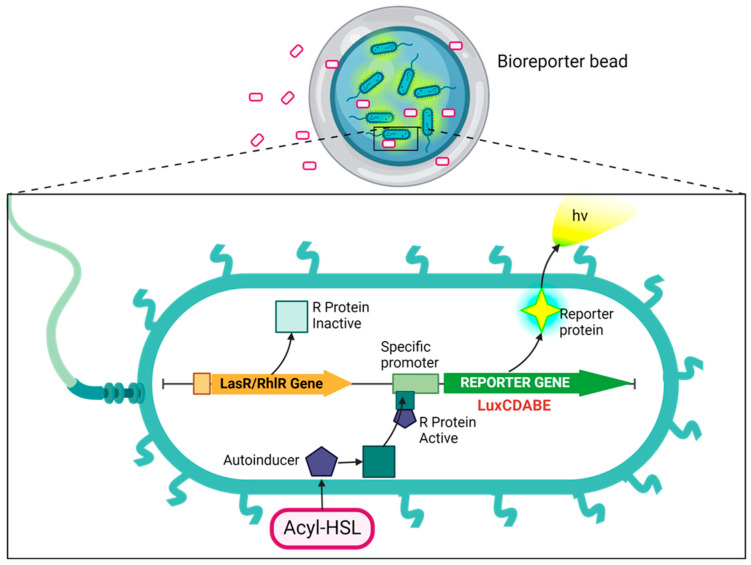
Schematic of the cellular events that result in the expression of a reporter protein. The cell can easily take up a bioavailable analyte, such as AHL, through its membrane. AHL (3-oxo-C_12_-HSL and C_4_-HSL) binds with a regulatory protein called LasR or RhlR, forming a protein–AHL complex. This complex binds to its corresponding promoter, *Plasl* or *Prhl*, which triggers activation of the transcription and translation of a reporter gene called luxCDABE. This results in the expression of the reporter protein, luciferase, inducing the bioluminescent reaction characterized by the emission of blue-green light (~490 nm). Created with BioRender.com, accessed on 13 June 2024.

In this work, the bioluminescent reporter gene (*luxCDABE*) is fused downstream of the luxR-promoter. Among cell-based biosensors, the *luxCDABE* reporter is reputed for the lowest detection limits (down to 10^−18^ mol) it offers [13]. The luxA and luxB genes within the luxCDABE gene cassette encode for bacterial luciferase, while the luxC, luxD, and luxE genes in the same cassette encode for the enzymes required for the synthesis and recycling of the luciferase substrate. This setup eliminates the need for any external addition of the substrate, enabling continuous real-time monitoring with low background interferences [12]. Transcription is initiated by binding the AHL–luxR complex to the promoter, leading to concomitant luminescent production (Figure 1). The amount of luminescence generated can be correlated with the concentration of the standard AHLs from which the calibration curve is generated. This can be used to quantitatively determine the presence of the quorum-sensing molecules in test/unknown samples. Our research advanced the development of whole-cell sensing systems by immobilizing them through encapsulation in reinforced alginate beads. These beads can be utilized as reporter microbeads for monitoring the AHLs in biological and environmental samples, including culture supernatants and biofilm flowthroughs. Whole-cell biosensing systems possess attributes that make them ideal for portable field kits. These attributes include the capacity to withstand a wide range of environmental conditions (such as various temperatures, pH levels, and ionic strengths), providing information on analyte bioavailability and requiring minimal or no sample pretreatment. Additionally, they are highly sensitive, selective, are easily prepared, provide rapid detection, are cost-effective, and are amenable to high-throughput screening and miniaturization [14].

Hydrogels are water-retaining and water-insoluble three-dimensional cross-linked polymeric networks. Sodium alginates are naturally occurring polyanionic polysaccharides comprising a linear copolymer of 1 → 4-linked β-D-mannuronic acid (M units) and α-L-guluronic acid (G units). Sodium alginate possesses a unique ability to chelate with multivalent cations, such as divalent ions like Ca^2+^ and Ba^2+^ in solution via the carboxylic acid groups of the G-G blocks, to form insoluble calcium alginate with an “egg-box” molecular structure [15]. Owing to this rapid and nontoxic preparation approach, alginate has emerged as a choice biomaterial for various encapsulation applications [16,17]. Calcium alginate hydrogel is unstable in physiological solutions and the presence of cation scavengers such as citrate, ethylene diamine tetra acetic acid, and phosphates. The calcium ions can, therefore, be exchanged by other ions, resulting in a loss of cross-links from the alginate gels. This makes the gels structurally and mechanically weaker, leading to uncontrollable permeability and eventual loss of the stable 3D structure [15]. This effect has continued to limit the widespread applications of calcium alginate hydrogels. Therefore, to address the above problem, efforts have been made to increase the chemical and physical durability of alginate gels by coating them with cationic and anionic polymers such as chitosan, as well as by exploiting several covalent cross-linking strategies and a composite-based approach [15,18]. To that effect, in this study, poly-lysine-reinforced calcium alginate was used to encapsulate the bioreporter in a two-step approach. Although Strand et al. 2003 employed confocal laser scanning microscopy (CLSM) to characterize the microcapsules of alginate-poly-L-lysine in terms of layer formation, stability, and distribution [19], the biotechnological applications of these capsules have been sparingly employed in drug release experiments [16] and cell encapsulation studies [20].

**Figure 2:**
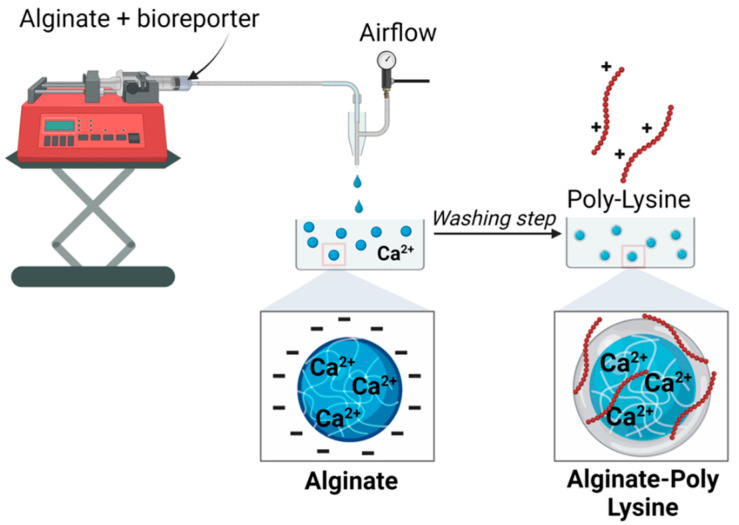
Schematics of the microcapsule synthesis. The size of the beads can be controlled by changing the flow rate of the feed solution (alginate + bioreporter) and the air pressure.

This work aimed to develop an alginate-based bioreporter encapsulation for detecting quorum-sensing molecules with a rationally designed coating developed through poly-lysine assembly. In brief, our approach involves sending a feed solution of alginate and engineered *P. aeruginosa* (PAO-JP2) strains through a microcapillary (which is connected to an airstream) into a reservoir containing Ca^2+^ to form calcium alginate microbeads (Figure 2). The resulting PAO-JP2-alginate microbeads were incubated in a PLL solution, resulting in a core–shell structure with a core of alginate that is cross-linked by Ca^2+^ and a thin shell of PLL that forms electrostatic interactions between the carboxyl of alginate and the ε-amino groups of PLL. The thin polymer shell around the alginate core stabilizes the overall microcapsule. Even if the alginate core became degraded due to ion exchange or chelation (in citrate or other solution), the microcapsule remains intact thanks to the PLL membrane.

The above procedure was used to encapsulate bioreporter bacteria and allow the free passage of exogenously added small quorum-sensing (QS) molecules that elicit a dose-dependent biochemical response from the bioreporter (Figure 1). The prepared bioreporter beads demonstrated biosensing ability toward both synthetic and secreted autoinducers. Furthermore, the bioreporter beads effectively detected the presence of a synthetic quorum-sensing inhibitor, furanone C-30. These beads exhibited excellent storability in all tested conditions, requiring no additional preparation before use.

**Figure 3:**
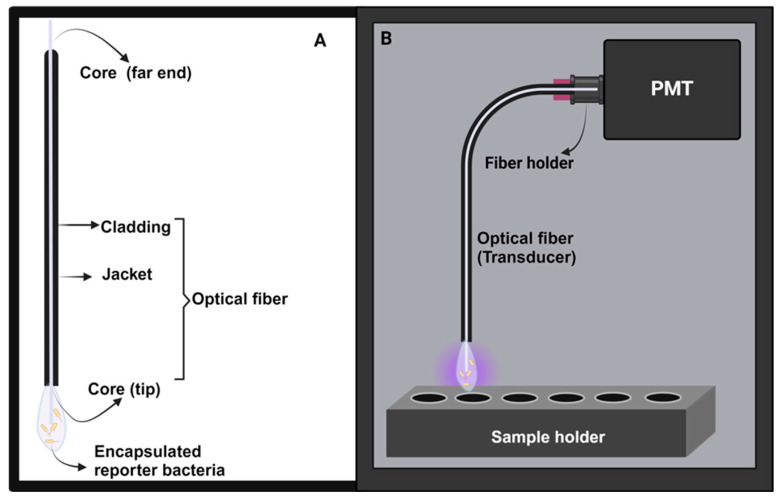
The basic components of the whole-cell, fiber-optic biosensors. (**A**) Optical fiber with a bioreporter immobilized unto the core. (**B**) Light–tight portable black box encasing all the biosensor components. For the image of the black box, readers are directed to the Supplementary Materials section (Figure S19).

As a proof of concept, the bioreporters in this study were employed to develop optical fiber-based, whole-cell biosensors using the established optical setup developed in our laboratory [21,22,23,24]. The encapsulated bioreporters serve as the recognition element, the optical fiber as the transducer, and the photomultiplier tube as the detector in a compact, light–tight ‘black box’ (Figure 3 and Figure S19). This makes the microbeads suitable as an on-demand capsule for potential wide application in monitoring and diagnostics.

## 2. Materials and Methods

### 2.1. Materials

Sodium alginate (low viscosity, A2158), trimethoprim, poly-l-lysine, poly-D-lysine, calcium chloride, Luria–Bertani medium (LB broth), and LB agar were purchased from Becton (Dickinson & Company, Le Pont de Claix, France). Acyl-homoserine lactone (acyl-HSL) 3-oxo-C_12_-HSL, C_4_-HSL, C_6_-HSL, 3-oxo-C_8_-HSL, 3-oxo-C_6_-HSL, and (as in Figure S2) the stock (5 mM) solutions were stored at −20 °C until required, and, as well as QS inhibitor—furanone C-30, were purchased from Sigma-Aldrich (St. Louis, MO, USA). Difco Luria–Bertani (LB) broth, Miller (10 g of L^−1^ tryptone; 5 g of L^−1^ yeast extract; and 10 g of L^−1^ NaCl) and Difco LB agar, and Miller agar (10 g of L^−1^ tryptone; 5 g of L^−1^ yeast extract; 10 g of L^−1^ NaCl; and 15 g of L^−1^ agar) were obtained from Becton (Dickinson & Company, Le Pont de Claix, France). The LIVE/DEAD^TM^ BacLight^TM^ Bacterial Viability Kit was obtained from Invitrogen, Thermo Fisher Scientific, Waltham, MA USA.

#### Bacteria Strains

LasR and RhlR reporters—*P. aeruginosa* PAO-JP2-luxCDABE and PAO-JP2 (pKD-rhlA) QS reporters.

PAO-JP2 (pKD201-*lasI*) is a double mutant (lasI/rhlI-deleted) strain of *P. aeruginosa* PAO1. It harbors a plasmid pKD201 with a lasI promoter coupled upstream to the luxCDABE luminescence system operon, which is responsive to 3OC12-HSL for LasR activity detection, as described previously by Ganin et al. [25]. PAO-JP2 (pKD-rhlA) is a lasI*-rhlI* double mutant of *Pseudomonas aeruginosa* PAO1 that harbors a pKD vector with *a rhlA* promoter coupled upstream to the luxCDABE operon. K. Duan et al. [26] developed the reporter strain responsive to C_4_-HSL (Figure S1). The bacteria strains were obtained from Prof. Michael Meijler, M. M. Ben-Gurion University of the Negev, Be’er Sheva, Israel. The scheme of the plasmid is presented in the Supplementary Materials, Figure S1.

### 2.2. Synthesis of Microcapsules

The microbead synthesis device was fabricated similarly to what Ahn et al. described [27] The feed solution was 2% (*w*/*v*) sodium alginate, and the reservoir was made of 150 mM of CaCl_2_ in water. The feed was loaded into a syringe and fed through the capillary device mentioned above, with the feed flow rate typically being 30 mL/h. The feed solution was converted into droplets using an air-driven droplet generator (Figure 2). As previously described, droplets were sheared off from the capillary tip by pulses of compressed air into an unstirred reservoir solution; after that, they were cross-linked into capsules. The capsules were allowed to cross-link for 10 min, after which they were washed and stored in double-distilled water (DDW) for subsequent steps.

#### 2.2.1. Bacteria Strain Growth Conditions

All cells involved in encapsulation and characterization experiments were PAO-JP2 (pKD201-*lasI*) and PAO-JP2 (pKD-*rhlA*), abbreviated as LasR and RhlR, respectively. The glycerol stocks of reporter strains PAO-JP2 (pKD201-*lasI*) or PAO-JP2 (pKD-*rhlA*) were stored at −80 °C. The stocks were streaked on to an LB agar plate containing 300 µg/mL of trimethoprim (TMP), incubated at 37 °C overnight in an incubator (Binder, Camarillo, CA, USA) to form colonies, and then stored in the refrigerator for less than a month. Starter cultures of LasR or RhlR were prepared by individually introducing a single colony into 10 mL of LB (Difco Lenox medium, BD, Le Pont de Claix, France) broth, supplemented with 300 µg mL^−1^ of TMP (Sigma-Aldrich, MO, USA), grown overnight in a shaking incubator (New Brunswick™ Innova^®^ 44/44R Shaker) at 37 °C, and shook at 220 rpm to attain an exponential growth phase. The fast-growing bacteria were reinoculated into fresh LB broth in a 1:50 dilution and grown to an approximate optical density of 0.6–0.9 at 600 nm (OD_600_). Then, a determined volume of the bacteria culture was pelleted in a sterile 50 mL tube by centrifugation at 4 °C and 3000 rpm for 15 min. The supernatant was discarded while the cell pellet was resuspended in 1000–2000 µL of LB and refrigerated until use.

#### 2.2.2. Encapsulation of Bacteria in Single-/Multiple-Layer Microcapsules

Poly-L/D-lysine-stabilized, bacteria-loaded alginate microbeads were prepared using a simple two-step approach similar to the method used in [28]. First, 1 mL of the diluted bacteria was added to a 4 mL portion of 2.5% *w*/*v* alginate solution (resulting in 2% *w*/*v* alginate solution), and this was then mixed well using a revolver tube rotator. The alginate bacteria solution was extruded into 150 mM of CaCl_2_ under a controlled extrusion flow rate and air pressure using the above-described capillary setup (Figure 2), and the resulting microbeads were allowed to harden for 10 min and rinsed with sterile double-distilled water (DDW). The microbeads were incubated in 2 mL of a 0.05% *w*/*v* solution of PLL (or PDL) for 10 min to allow the formation of a thin layer around the microbead, followed by washing with DDW. The resulting bacteria-alginate/PLL microbeads were subjected to 10 min incubation in 0.05% *w*/*v* alginate to form an outer layer on the capsules and to neutralize the non-reacted PLL, creating a negatively charged surface, and then rinsed again. This standard encapsulation approach was adopted in all subsequent experiments unless otherwise stated.

For double-membrane microbeads, the same procedure was followed as for the single-membrane microbeads, except that the PLL incubation time was 7.5 min. After the alginate step, the microcapsules were incubated for 7.5 min in 2 mL of 0.05% PLL, which were then rinsed, incubated in 0.05% alginate for 10 min, and then the rinsed again, resulting in double-membrane microbeads.

The triple-membrane microbeads were prepared in the same way as the double-membrane microbeads. After the 0.05% *w*/*v* alginate step, the double-membrane microbeads were incubated in 2 mL of 0.05% *w*/*v* PLL for 5 min and washed. The microbeads were then coated again with a 0.05% *w*/*v* alginate solution. The size (diameter) of the beads was determined using a standard bright-field microscope, a 2.5× objective lens, and ImageJ 1.54f. Alginate-poly-lysine and alginate-alone beads were tested for stability in the presence of a Ca^2+^ ion chelator and sodium citrate.

### 2.3. Structural Characterization

#### 2.3.1. X-ray Photoelectron Spectroscopy (XPS) Analysis

X-ray photoelectron spectroscopy (XPS) analysis was conducted on the dehydrated microcapsules, beads, alginate, and PLL films using an “ESCALAB Xi+” instrument by Thermo-Fisher Scientific. The ionization energies of the various elements present in the samples were measured. The analysis was performed under an ambient pressure of <1 × 10^−10^ mbar. Aluminum Kα radiation source (1486.68 eV) was used for photoelectron emission at room temperature, and the spectra were collected at a 90° angle from the X-ray source. A low-energy electron flood gun was employed to minimize surface charging, and the measurements were taken with a spot diameter of 650 µm. Spectral analysis was carried out using the Avantage software version 6.6.0 provided by Thermo-Fisher Scientific. High-resolution spectra of C1s, O1s, N1s, and Ca1s peaks and a survey spectrum at 20 eV pass energy were obtained.

#### 2.3.2. Attenuated Total Reflectance-Fourier Transform Infrared (ATR-FTIR) Analyses

The outer portion of alginate-PLL microcapsules was examined using Smart iTR™ Diamond ATR-FTIR sampling accessory and Nicolet 6700 spectrometer to examine the interactions between the molecules within the alginate-PLL microcapsule membrane. All measurements were taken on the built-in diamond ATR crystal. The FTIR spectra were recorded over the range of 4000–650 cm^−1^ at 4 cm^−1^ resolutions and represented an average of 36 scans. The spectrum of the clean dry diamond ATR crystal in the ambient atmosphere (air) was used as the background for infrared measurement.

#### 2.3.3. Scanning Electron Microscopy (SEM)

Scanning electron microscopy was performed on the double-layer hydrogels (freeze-dried to maintain the porous structure without any collapse) to obtain information on the external structure of PLL-coated and non-coated alginate hydrogels. The sufficiently swollen hydrogels were removed from the solution, quickly frozen at −86 °C for 2 h, and then freeze-dried under a vacuum at room temperature for 3 days to remove the imbibed water completely until the samples became completely dry prior to morphological observation. The freeze-dried hydrogels were mounted onto the base plate and coated with gold (~10 nm). The morphology of the hydrogels at room temperature was observed using a field-emission scanning electron microscope (FESEM, Thermo Fisher Verios 460L) at different magnifications.

### 2.4. Bioluminescent Assay

The bioluminescent response of the alginate-poly-lysine immobilized bioreporter PAO-JP2 (pKD201-*lasI*) and PAO-JP2 (pKD-*rhlA*) strains were acquired throughout a 20–30 h period using the Luminoskan Ascent Luminometer (Thermo Fisher Scientific, Waltham, MA, USA) and were maintained at 37 °C. The luminometer was set to a kinetic mode at 5 or 10 min intervals between readings. White (non)-transparent, 96-well, and flat-bottom microplates (Nunc, Roskilde, Denmark) was used in all experiments. The experiments were carried out in three/four biological replicates with at least two independent experiments. The maximum luminescence values (expressed in the relative luminescent unit, RLU) were extracted using Microsoft Office Excel, from where the induction factor was calculated as a ratio of the maximum RLU of the test to that of the negative control (in the absence of an inducer).

#### 2.4.1. Viability Assay of the Immobilized Bacterial Strains

Microcapsules containing LasR and RhlR strains were placed in a sterile 24-well plate containing LB broth and were mildly shaken for about 25 h in an orbital shaker in a hot room maintained at 30 °C. For live/dead staining, capsules were washed with saline solution (0.9% *w*/*v* NaCl) three times. After washing, capsules were incubated at room temperature for 15 min in a saline solution containing a propidium iodide (dead) and SYTO™ 9 (live) stain washed with saline, and these were then transferred to an ibid 12-well plate for confocal microscope imaging.

#### 2.4.2. The Detection of Synthetic AHLs (3-oxo-C_12_-HSL and C_4_-HSL)

The ability of the immobilized reporter bacteria to detect the sub-nanomolar concentration of the inducer was assessed by incubating the bioreporter microbeads in the presence of a known concentration of the inducers.

Typically, 3–10 microbeads were added to each well in a white non-transparent, 96-well, and flat-bottom microplate (Nunc, Roskilde, Denmark), followed by the addition of 190 µL of LB broth (containing 300 µg/mL of Trimethoprim) incubated at 37 °C with/without shaking at 220 rpm for a specified period. Dedicated calibration curves were obtained by adding 10 µL of the QS molecules to the well containing the microbeads at a range of final concentrations, 5 µM–500 pM of 3-oxo-C_12_-HSL (for the LasR reporter), and 10 µM–100 pM of C_4_-HSL (for RhlR reporter). The plate was incubated for 20–30 h at 37 °C, and, during this time, luminescence measurements were performed at 5 or 10 min intervals using a Luminoskan ascent multi-mode reader luminometer (Thermo Scientific, Shanghai, China) and another luminometer (Luminoskan, Thermo Fisher Scientific, Waltham, MA, USA). The equations derived from the calibration curves were used to calculate the concentration of the 3OC_12_-HSL and C_4_-HSL present in both culture supernatant and biofilm flow-through samples. All the experiments were carried out in three or four replicates and repeated at least twice.

#### 2.4.3. Detection of AHLs in the Supernatant of Wild Cultures of PAO1 Strains

A bioreporter was employed to detect the presence of the QS molecules in the supernatants of the culture of a wild strain of *P. aeruginosa*. The overnight culture of PAO1 was briefly reinoculated and grown overnight in a shaking incubator (New Brunswick™ Innova^®^ 44/44R Shaker) at 37 °C and shook at 220 rpm until the OD_600_ was about 0.9. Then, a determined volume of the bacteria culture was pelleted by centrifugation at 4 °C and 10,000× *g* for 10 min. The supernatant was filtered through a 0.22 µm-membrane filter and used as a source of AHLs for the biosensor testing.

#### 2.4.4. The Detection of AHLs in the Biofilm Effluent Obtained in the Flow-Cell Experiment

The biofilm experiment was carried out under flow conditions using a standard method, as shown in the following Supplementary Materials scheme (Protocol S1 and Figure S12) according to Goldberg et al. [29]. The flow was allowed to continue undisturbed for 12, 24, 48, and 72 h, and the flow through (effluent) at the determined time was collected, filtered using a 0.22 µm-membrane filter, and stored at −80 °C until required. The flow through was used as the source of AHLs for the biosensor testing. As stated in the Supplementary Materials section (Protocol S2), the resulting biofilms were macroscopically visualized using CLSM and were 3D-processed by IMARIS 2.7 software (Bitplane AG, Zurich, Switzerland).

#### 2.4.5. Quorum-Sensing Inhibition Assay

The ability of the biosensor to detect quorum-sensing inhibitors was evaluated by incubating the bioreporter microbeads with various concentrations of a synthetic quorum-sensing inhibitor (furanone-C-30) in the presence of a known concentration of autoinducers or cell-free supernatants of the wild *P. aeruginosa* strain (PAO1). The bioluminescent measurement was carried out as usual, and the percent inhibition was determined according to the following equation:

$$\mathrm{Quorum-sensing}\;\mathrm{inhibition}\;(\%)=\frac{Induction\;Factor\;in\;the\;presence\;of\;Inhibitor}{Indcution\;Factor\;in\;the\;Absence\;of\;Inhibitor}\times 100.$$

### 2.5. Development of Optical Biosensors

As a proof of concept for biosensor development, the bioreporter hydrogels were adapted to the fiber optic setup established in our laboratory [21,22,23,24]. The set up comprises the biological recognition elements (LasR and RhlR bioreporter strains) attached to a transducer (fiber optic) whose far end is in contact with the detector (photomultiplier tube), as shown in Figure 3.

#### 2.5.1. Instrument Setup

The photon-counting system was designed and built in our laboratory. The instrument setup was placed in a light-tight box (Figure 3). A manual shutter (71430, Oriel) was placed in front of the detector to prevent damage to the photon-counting unit by environmental light [23]. The analog output signal was the mean of the AC components (pulses) generated after multi-anode amplification in the photomultiplier tube (PMT). The bioluminescence measurements at 490 nm were recorded using a Hamamatsu HC135-01 PMT Sensor Module, which combines the sensitivity of a photomultiplier tube with the advanced capabilities of a microcontroller [21]. The detector was optimized to the blue light region and includes a 21 mm-diameter active area convenient for gathering light radiation without any optical-focusing elements [23].

#### 2.5.2. Experimental Probe Setup

The optical fiber, with its adsorbed bioreporter, was placed in a 0.5 mL conical tube (Jonplast, Lesmo, MB, Italy) containing the test sample solution and was held at the epicenter of the light-proof sample holder to avoid ambient light interferences. The far end of the fiber was held by a fiber holder (FPH-DJ, Newport) and placed into an adjustable single-fiber mount (77837, Oriel).

#### 2.5.3. Preparation of the Optical-Fiber Surface and Bioluminescent Measurements

Multimode optical fibers, PUV 400 BN (CeramOptec, GmBH, Bonn, Germany), were used in these experiments. They are made of a pure silica core diameter of 400 μm, with a refractive index of 1.4571 (at 633 nm) and a cladding diameter of 440 μm with a refractive index of 1.4011 (at 633 nm). The black nylon jackets were stripped away from a 1 cm long optical-fiber tip, which was then used for the immobilization of bioreporter strains [21,22].

The 1 cm-optical-fiber tip was exposed to a bacterial alginate suspension and then dipped into a calcium chloride solution, entrapping the bioreporter within a hardened calcium alginate matrix. Repeating these steps six (6) times resulted in a thick adlayer, increasing the number of bioreporters immobilized on the optical-fiber transducer [22]. At this step, the functionalized optical fibers were ready for the experimental detection of autoinducers. Fibers were used immediately after preparation.

### 2.6. Statistical Analysis

A two-way or one-way ANOVA, followed by a Tukey multiple comparisons test, was conducted using GraphPad Prism version 8.0.2 for Windows (GraphPad Software, San Diego, CA, USA, www.graphpad.com, accessed on 26 November 2023).

## 3. Results

### 3.1. Encapsulation and Optimization

#### 3.1.1. Optimization of Microbead Formations

Using the experimental setup described earlier, it was possible to control the bead’s size by controlling the microfluidic pump’s air pressure and flow rate. At a constant flow rate of 10 mL/h, the higher air pressure (valve opening) produced correspondingly smaller-sized beads until 40 psi (Figure S3). Much smaller beads were obtained when the flow rate was tripled (30 mL/h) at a constant air pressure of 40 psi, resulting in an average bead diameter of 956.23 ± 38 µm. Therefore, a 30 mL/h flow rate, 40 psi pressure, and 15-unit aperture were adopted in all the subsequent experiments.

#### 3.1.2. Effect of Encapsulation Methods

Next, our research investigated the encapsulation of the bacteria in the microbeads. The process of encapsulating bacteria within alginate presents challenges due to potential gel degradation from ion exchange or rapid bacteria growth, which could lead to bacteria escaping from the gel matrix [30]. To address this issue, the polymer shell surrounding the alginate core can serve to protect the cells and maintain them within the core. It is important to note that the polymer shell allowed for the diffusion of small molecules in and out of the core, which is crucial for maintaining cell viability.

Two approaches were investigated to prepare a poly-lysine-reinforced alginate microbead encapsulation system. The first approach involved preparing an alginate inner sphere followed by stepwise incubation in a poly-lysine solution to create a thin membrane around the alginate (Figure 2). In the second approach, the microbeads were dropped into a calcium-poly-lysine solution, followed by two additional incubations in a PLL solution (Figure S4). Both approaches led to reinforced alginate microbeads that are stable when incubated in the presence of 5% sodium citrate.

#### 3.1.3. ATR-FTIR Data

**Figure 4:**
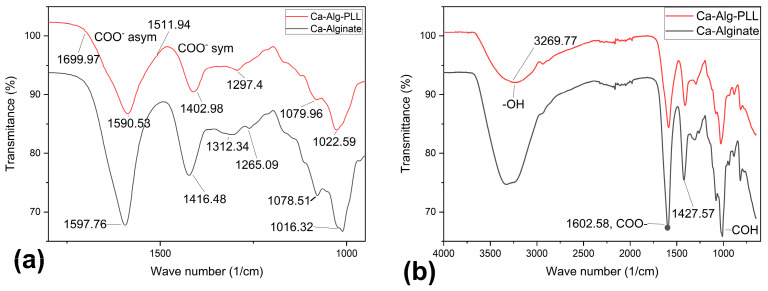
The ATR-FTIR absorbance spectra of the outer dehydrated alginate-PPL microcapsules. (**a**) The 1700–900 cm^−1^ region of the spectra that corresponds to the asymmetric and symmetric stretching vibrations characteristics of the carboxyl (COO^−1^) functional group. (**b**) The full spectra of ca-alginate, alginate-PLL, and the characteristic peaks are discussed in the text. A spectrum of the alginate/PLL interpenetrating network and sodium alginate can be found in the Supplementary Materials section (Figure S5).

Figure 4 compares the ATR-FTIR absorbance spectrum of the outer portion of lyophilized alginate-PLL and calcium-alginate microbeads. The absorbance bands that were characteristic of the alginate were identified with a broad peak around 3304 cm^−1^, which were ascribed to the stretching vibrations of the hydrogen-bonded OH groups and the two peaks around 1590 and 1416 cm^−1^ representing asymmetric and symmetric stretching vibrations, respectively. Absorbance peaks at around 3265, ~1645, and ~1540 cm^−1^ were ascribed to the Amide A, Amide I, and Amide II, respectively, of PLL [31,32]. The amide bands arose from the stretching and bending vibrations of the N-H bonds and stretching vibrations of the C=O and C-N bonds of the amide group in the PLL [32]. As the distinguishing peaks of PLL coincided with the major peaks of alginate, as shown in Figure 4, the spectrum of alginate-PLL appeared like the spectrum of alginate except in the band intensity.

#### 3.1.4. XPS Analyses

**Figure 5:**
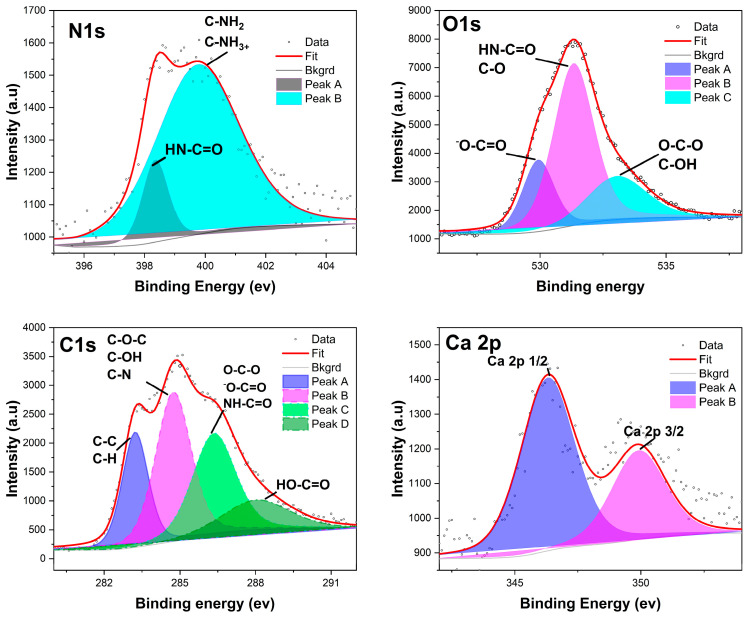
The deconvoluted XPS spectrum peaks of the lyophilized alginate-poly-lysine beads. The deconvoluted peaks were assigned to a chemical group based on the binding energy of the peaks (N_1s_, O_1s_, C_1s_, and Ca_2p_), as shown in Figure S6.

XPS was applied to quantitatively determine the elemental and chemical composition of the outermost portion of the alginate-poly-lysine microcapsules. This technique is surface-sensitive in that only a sample’s outermost (about 10 nm) region is analyzed [31]. High-resolution spectra were recorded for oxygen, nitrogen, and carbon, as shown for poly-lysine in Figure 5. The samples’ surface elemental compositions (expressed as relative atomic percentages) were computed from the XPS spectra recorded in survey mode (Figure S6). Nitrogen, however, is only a minor component of not more than 10.7% of the total PLL molecule [33]. The actual difference between alginate and alginate-poly-lysine can be calculated by dividing the N/C ratio of PLL by the N/C ratio of the capsule membrane. This calculation shows that alginate-PLL membranes are composed of 56% PLL and 65% PDL (Figure S6).

The survey surface analyses of alginate and alginate-PLL revealed the elemental composition of alginate-PLL to be composed of 58.63% carbon, 33.39% oxygen, 5.85% nitrogen, and 1.58% calcium. While the uncoated alginate showed a similar composition of carbon and oxygen (56.51% C and 36.57% O), uncoated alginate has a higher composition of calcium (4.48%, Ca) than the PLL-coated alginate (Supplementary Materials section, Figure S6). This is probably because XPS is a surface-sensitive analysis that is conducted within a 10 nm depth.

#### 3.1.5. Scanning Electron Microscopy (SEM)

**Figure 6:**
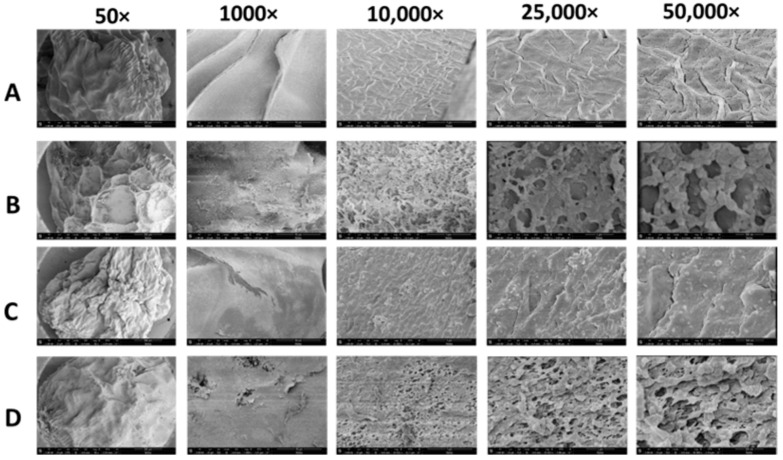
Scanning electron microscopy (SEM) images of the outer part of the alginate and alginate-poly-lysine microbeads at different magnifications. (**A**) Alginate microbeads prior to the poly-lysine (PLL) coating. (**B**) Alginate-PLL microbeads. (**C**) The alginate/PLL interpenetrating network (IPN) prior to the PLL outer coating. (**D**) PLL-coated alginate/PLL IPN.

The morphology and composition of the freeze-dried beads were examined using FE-SEM at different magnifications. Notably, the poly-lysine-coated beads (Figure 6B,D) exhibited distinct morphology compared to the uncoated alginate and IPN of alginate/PLL (Figure 6A and Figure 6C, respectively) at varying magnifications.

#### 3.1.6. Microbead Stability and Swelling

**Figure 7:**
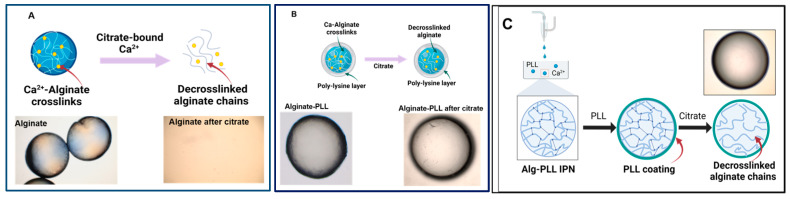
Stability of alginate-poly-lysine microbeads in the presence of cation scavengers. (**A**) The alginate microbeads (control) were rapidly degraded within 60 min when incubated in 5% *w*/*v* sodium citrate because of the Ca^2+^ removal from the hydrogels. (**B**) Alginate-PLL. (**C**) The alginate-PLL IPN capsules remained stable in a 5% sodium citrate solution after 14 h.

The main reason for adding a poly-lysine shell around the alginate gels was to prevent their degradation when they came in contact with certain ions or chelators. Therefore, the microbeads of bare alginate and alginate–poly-lysine were placed in 5% sodium citrate, a well-known chelator of Ca^2+^ ions. Figure 5 shows the result, which depicts the scheme of the alginate and PLL-coated beads incubated in a citrate solution. As expected, the bare alginate microgels completely degraded within 60 min (Figure 7A).

As seen in the schematics, the degradation occurred because citrate removes Ca^2+^ cross-links, leaving linear alginate chains that are no longer part of a 3D network (Figure 7). In contrast, alginate–PLL and alginate–PDL microbeads retained their sphericality in the citrate solution (Figure 7B). This result shows that, regardless of any loss of cross-links from the alginate core, the polymer shells ensure that the microcapsules preserve their structural integrity, although the resulting alginate-PLL microbeads treated with citrate were found to be weaker than their untreated counterparts.

**Figure 8:**
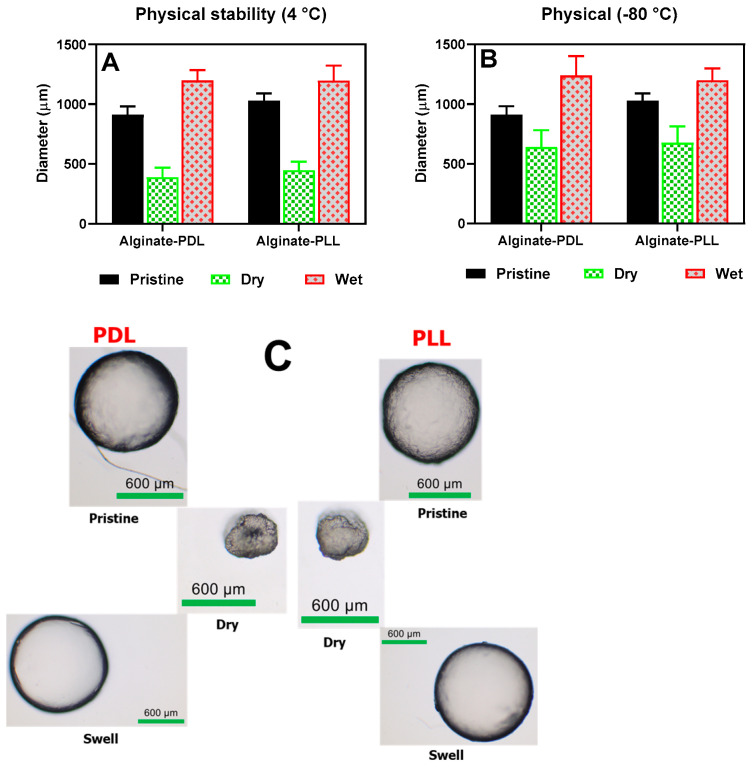
Swelling properties. (**A**) beads stored in refrigerators (pristine), air-dried, and rehydrated (wet). (**B**) beads stored at −80 °C (pristine), air-dried and rehydrated (wet). (**C**) The physical appearance of the beads during wet, dried, and swelled conditions.

The beads’ morphological appearances and sizes were determined under various treatment conditions, such as freezing, drying, and rehydrating, as shown in Figure 8. Poly-L-lysine- and poly-D-lysine-coated alginate beads behaved alike in all the conditions tested (frozen, dried, and rehydrated). The beads shrank into irregular, smaller shapes upon drying, and then full sphericity was restored after rehydration in about 30 min (as shown in the upper frame in Figure 8C).

### 3.2. Biological Activity Tests

#### 3.2.1. Viability and Bioluminescence

**Figure 9:**
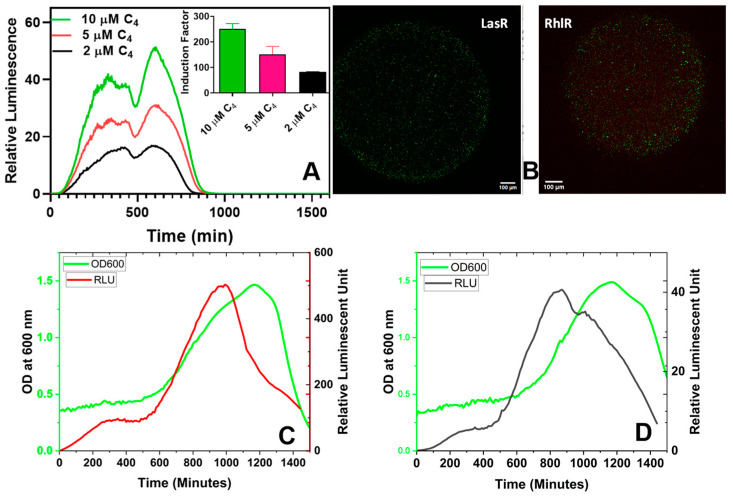
The bioluminescent response of the immobilized RhlR in different C_4_-HSL concentrations. The insert in (**A**) illustrates the induction factor calculated using the maximum relative luminescence unit (RLU) derived from the spectral data. The induction factor is the ratio of the test’s maximum RLU conducted in the presence of an inducer to the maximum RLU obtained in the absence of added inducers. (**B**) Confocal images of the live/dead stained bacteria within alginate-PLL beads. (**C**) Both the luminescence and cell density (absorbance at 600 nm) of the LasR bioreporter. (**D**) The luminescence and OD_600_ readings of the RhlR bioreporter. The OD_600_-normalized luminescence reading (RLU/OD_600_) can be found in Figure S8.

**Figure 10:**
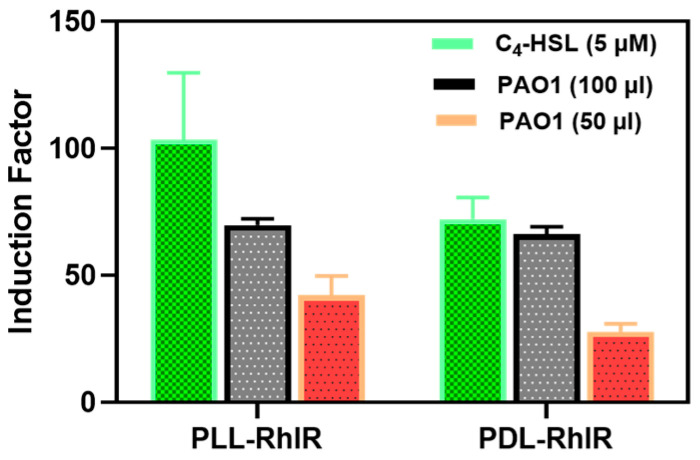
The bioluminescent responses of the PLL- and PDL-coated alginate were similar. The PDL and PLL coatings were compared regarding the bacteria’s response to the added synthetic (C_4_-HSL) and secreted (by the PAO1 wild strain of *P. aeruginosa*) autoinducers.

The live/dead bacterial viability stain is a fluorescence-based approach used to monitor the bacteria population based on the integrity of the cell membrane. Cells with compromised membranes that are dead or dying are permeable to propidium iodide, which will stain red with propidium iodide. In contrast, cells with an intact membrane will stain green with SYTO™ 9. Figure 9B confirms the viability of the immobilized bacteria strains alive within the alginate-PLL beads (Figure S7). Also, the encapsulated bioreporters demonstrated a dose-dependent bioluminescent response to the exogenously added autoinducers (Figure 9A,C,D), showing that the bacteria were not only alive but also functional, and that the encapsulating microbeads were permeable to the added autoinducers (AIs). The bioluminescent response was not different (*p* < 0.05) between the bacteria cells encapsulated within the microbeads coated with PDL or PLL, showing that the microbeads were permeable toward the probed autoinducers (Figure 10).

#### 3.2.2. Bioluminescent Response of Alginate Reinforced with L- and D-Isomers of Poly-lysine

A possible alternative to PLL is the use of poly-D-lysine (PDL), a chiral form of α-poly-lysine, which is widely used in myriad biomedical applications [34]. While PLL is susceptible to proteolytic degradation [34], PDL is not because most biological enzymes do not recognize the D configuration on the D amino acid isomers [35,36,37]. This experiment is of interest in an application where what is being immobilized involves proteases protease as part of the physiological metabolism, or a scenario wherein the bioreporter microbeads might encounter protease in any unknown clinical or environmental samples. As a result, both isomers were independently used to reinforce alginate and tested for suitability in the bioassay tests. The results showed no significant difference (*p* < 0.05) between PLL and PDL regarding the physical features (Figure 8) and biosensor responses (Figure 10).

#### 3.2.3. Effect of Activation

**Figure 11:**
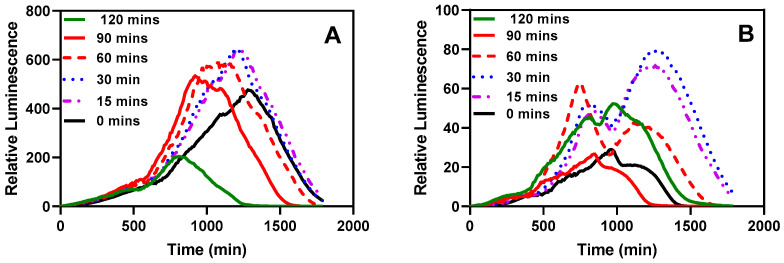
The effect of pre-incubation and shaking before testing. (**A**) Bioluminescent response of the LasR strain. (**B**) Bioluminescent response of the RhlR strain. The stored microspheres were analyzed with or without pre-incubation to determine the effect of pre-incubation on the biosensor performance of the reporter bacteria strains. Experiments were conducted with 0, 15, 30, 60, 90, and 120 min of incubation and shaking at 37 °C and 220 rpm. Each curve represents the mean of four replicate experiments.

One significant advantage of immobilizing bacteria through encapsulation is the capability to store them in a ready-to-use form, typically at lower temperatures. We aimed to investigate whether the bacteria would require a recovery period after being stored in the refrigerator for an extended duration. As depicted in Figure 11, an incubation period of 15–30 min at 37 °C with shaking at 220 rpm seemed optimal for recording bioluminescent readings. Even the sample without pre-incubation exhibited a noticeable response. A significant difference was observed in the time taken for the measurements to peak.

#### 3.2.4. The Construction of the Calibration Curves

**Figure 12:**
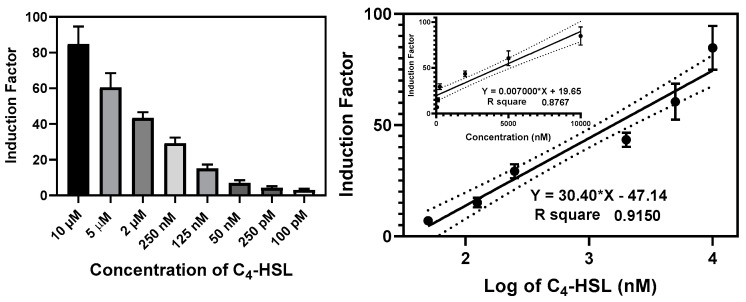
Calibration curve of the RlhR bioreporter beads at various concentrations of C_4_-HSL. The **left panel** shows the bioluminescence (expressed as an induction factor) produced by the encapsulated reporter in the presence of increasing concentrations of exogenously added C_4_-HSL. The **right panel** shows the dose–response relationships inferred from the data in the left panel. Linear regression lines (bold) were drawn on logarithmic and linear (upper insert) scales, and the 95% confidence interval limits were the black dotted lines parallel to the regression lines. The equation and the *R*^2^ value for each regression line are shown. Data are the mean ± SD of three independent experiments.

**Figure 13:**
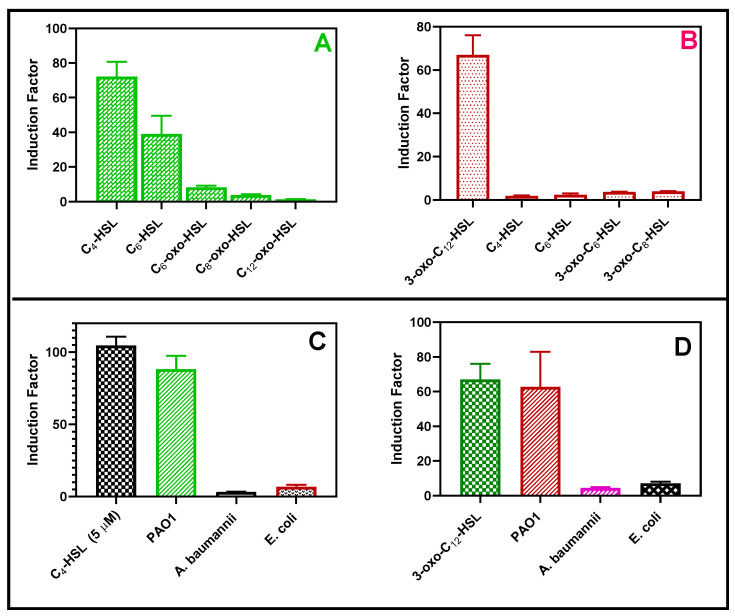
The bioreporter specificity test. The RhlR (**A**) and LasR (**B**) response toward 5 µM each of synthetic QS molecules, respectively. The second experiment was conducted with either 20 µL of cell-free supernatants of each Gram-negative bacterium such as Escherichia coli (*E. coli*), *Pseudomonas aeruginosa* (PAO1), and Acinetobacter baumannii (*A. baumannii*) (72 h biofilm set up), or 5 µM of C_4_-HSL for the RhlR strain (**C**) or 5 µM of 3-oxo-C_12_-HSL for the LasR strain (**D**).

The bioluminescent responses of the bioreporter beads were measured at various concentrations of the autoinducers for 20–24 h. The induction factor (IF) was calculated for each well, and the average induction factor of the replicates was plotted against the corresponding concentrations expressed in nanomolar or against the log of the concentrations (Figure 12 and Figure S9). The linear regression equation was generated to determine the 3-oxo-C_12_-HSL and C_4_-HSL present in the cell-free culture supernatant and biofilm effluent. The detection limit was set as the minimum autoinducer (AI) concentrations that elicit a luminescent response at ≥20% of the maximum obtained in an experiment without adding AIs. The RhlR-based bioreporter beads demonstrated a dynamic range and limit of detection of 10 µM–0.1 nM and 50 pM, respectively, for C_4_-HSL (Figure 13), while the LasR-based bioreporter showed a dynamic range of 5 µM–0.2 nM and a limit of detection of 0.1 nM for 3-oxo-C_12_-HSL (Figure S13).

### 3.3. Selectivity and Inhibition Studies

#### 3.3.1. Selectivity of the Bioreporters to the Synthetic and Secreted QS Molecules

**Figure 14:**
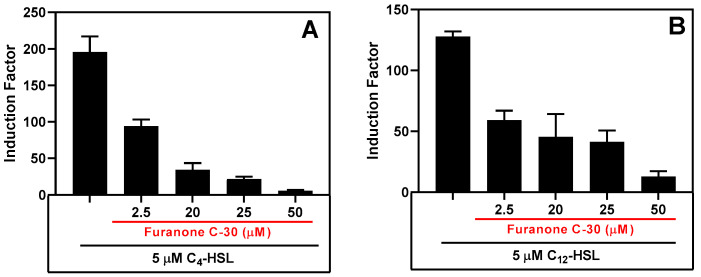
The QS inhibitory effect of furanone C-30 in the presence of synthetic AHLs. (**A**) The inhibition of RhlR signaling in the presence of 5 µM of C_4_-HSL and (**B**) the inhibition of the LasR bioluminescence system in the presence of 5 µM of 3-OC_12_-HSL. Luminescent readings were taken for 10 h, and the maximum relative light unit (RLU) was plotted. The results are the mean ± SD of biological triplicates.

The bioluminescent response of the bioreporter was tested in the presence of synthetic autoinducers (Figure 13A,B) and cell-free supernatants obtained from *Pseudomonas aeruginosa*, *Acinetobacter baumannii*, and *Escherichia coli* (Figure 13C,D). Each strain of the reporter bacteria in this study distinctly responded to their cognate autoinducers. The bioreporter selectively responded to the cell-free samples of *P. aeruginosa* but not *E. coli* or *A. baumannii*, as shown in Figure 14. This is desirable because the biosensor can selectively detect *P. aeruginosa*-specific autoinducers in the presence of other bacteria in the clinical and environmental samples.

#### 3.3.2. Determination of AIs in the Flow-Cell Biofilm Effluent

Biofilm formation was followed microscopically and as a function of autoinducer secretion, and it was confirmed by luminescence assays with RlhR and LasR bioreporters (Figures S13 and S14). The effluents of flow-cell biofilm were collected at 12, 24, 48, and 72 h intervals and filtered. The filtered effluents were used as the source of autoinducers, and the bioluminescence responses were recorded as usual. The bioluminescence was interpolated from the standard plot.

#### 3.3.3. Inhibition Studies

The biosensor’s ability to detect the presence of an inhibitor was tested by incubating it with either synthetic autoinducers (Figure 14) or cell-free supernatants of the wild PAO1 type in the presence of varying concentrations of QSI (Figures S15 and S16).

### 3.4. Storability Assessments

**Figure 15:**
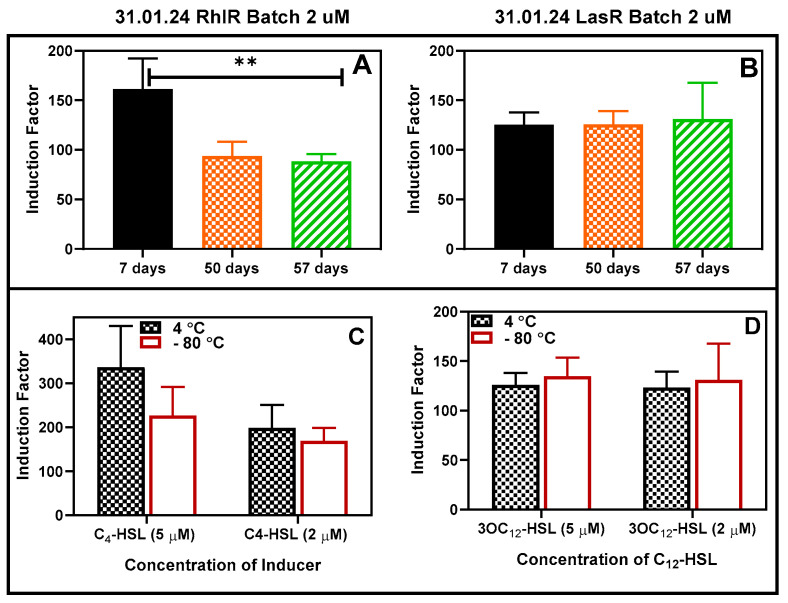
Storability of the bioreporter beads in refrigerator conditions. (**A**) RhlR and (**B**) LasR 2 µM inducers. The RhlR strain experienced a significant (shown as ** at *p* < 0.05) reduction in activities between Days 7 and 50 and remained fairly constant thereafter. Comparison of the effect of storage at 4 and −80 degrees (for 30 days) on the luminescence. The residual activity of RhlR (**C**) and LasR (**D**) was compared after storage in the refrigerator and freezer at −80 °C for a minimum of 30 days. Two concentrations of each AHL were tested, and the results are reported as the mean ± SD, n = 4. Statistical analysis revealed no significant differences in the readings at *p* < 0.05.

The bioreporter beads were tested for long storability under different temperatures. First, the bacterial beads were stored at −80 °C for 48 h, after which their activities were tested and compared with the corresponding beads stored at 4 °C (Figure S11). There was no statistically significant difference between the responses to the added autoinducers. The bioreporters were further stored at 4 °C for at least 57 days, and the activities were tested at intervals (Figure 15A,B). After that, the beads were stored at −80 °C and 4 °C for 30 days, after which the activities were tested and compared as usual and found to be the same at *p* < 0.05 (Figure 15C,D).

**Figure 16:**
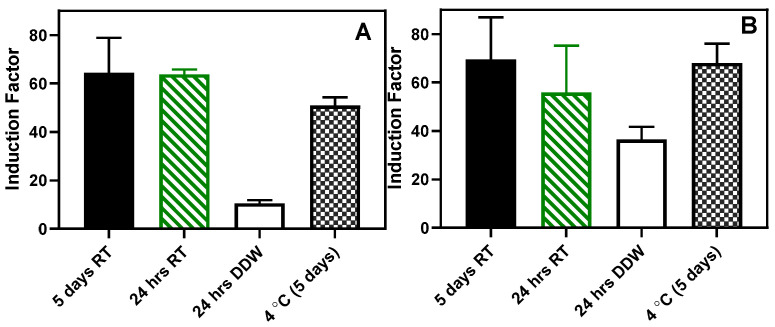
Storage at room temperature. (**A**) RhlR and (**B**) LasR results. The results are presented as the mean ± SD, (n = 4). RT: beads stored dried at room temperature.

Also, an attempt was made to store the beads at room temperature in dry and wet conditions. As shown in Figure 16, the beads stored in a dried state for 24 h and 5 days retained the sensing ability to the autoinducer without statistically significant differences (Figure 16). However, in both RhlR and LasR strains, when the beads were stored in sterile DDW for 24 h at room temperature, there was a significant (*p* < 0.05) loss in the sensing ability of the immobilized bioreporters. Similarly, when the beads were lyophilized, there was a considerable loss in the residual bioluminescent response (Figure S17).

## 4. Discussion

Monitoring quorum-sensing molecules (QSMs) has become a crucial analytical tool in researching bacteria-related disturbances. The levels of these signaling molecules can potentially serve as biomarkers for disease and environmental monitoring. Therefore, there is an increasing demand for analytical methods that can sensitively, quantitatively, rapidly, and cost-effectively detect bacterial QSMs. Whole-cell biosensing systems have been shown to detect QSMs directly with little or no sample pretreatment. Moreover, microorganisms are tolerant of suboptimal assay conditions and can be prepared in unlimited quantities relatively inexpensively, making them candidates of choice for high-throughput screening, miniaturization, and automation [38].

In this study, we postulated that the stable immobilization of bioreporter bacteria within reinforced alginate could offer robust whole-cell sensing systems for monitoring the levels of AHLs. First, poly-lysine-coated alginate beads were prepared, which remained intact in the presence of the known Ca^2+^ chelator citrate (Figure 7). The bacteria were retained within the beads, and the bioluminescent assays demonstrated the passage of autoinducers and quorum-sensing inhibitors into the core of the beads.

The alginate-poly-lysine capsules utilized in this research exhibited similar physical properties regardless of the type of poly-lysine (L and D) isomers and identical permeability to the QS molecules. This suggests that either L- or D-isomers of poly-lysine can be employed to reinforce alginate based on specific applications. For instance, the D-isomer is known to be less susceptible to proteolysis, making it suitable for the encapsulation of applications that are likely to encounter protease. For this study, the L-isomer of poly-lysine was utilized in all experiments. The physicochemical analyses presented clear evidence of the presence of alginate and PLL or PDL in the membrane of the APA microcapsules. The X-ray photoelectron spectroscopy (XPS) analysis of the microcapsules’ elemental composition indicated that relatively high amounts of PLL were very close to the surface, within the outermost being 10 nm. Tam et al. [31] and De Vos et al. [33,39] obtained similar surface compositions using XPS, implying that these results are predictable. Furthermore, the spectroscopic analyses indicated that both alginate and the PLL (or PDL) were present at the surface, suggesting that at least some of the polysaccharides bound to the PLL membrane during the capsule’s final incubation are diluted alginate. We excluded the possibility that the alginate at the surface originated from the gel core since XPS detected no calcium. Thu et al. also demonstrated that, using radiolabeling techniques, the coating alginate binds to the microcapsule’s PLL layer. Furthermore, the confocal microscopic study of the binding of PLL to the alginate revealed the formation of a polyanion/polycation outer, thin-shell-like, and rough membrane (Figure 6) similar to what was observed elsewhere [19,32]. The three-dimensional image of alginate-PLL capsules showed no visible holes in the PLL membranes, indicating that PLL covers the capsule completely. It was further demonstrated that the PLL–alginate interaction was unchanged when stored for two weeks and two years [19]. This shows the stability of the additional support provided by the PLL.

It was observed that, in this study, the PLL-coated alginate beads were protected from the effects of a known metal chelator, sodium citrate. Chelators such as EDTA and sodium citrate can induce the complete degradation of alginate gels, while the gels may undergo partial degradation when immersed in buffers like phosphate-buffered saline (PBS). This occurs as some Ca^2+^ cross-links are exchanged with Na^+^, reducing mechanical rigidity and cross-link density and ultimately weakening the gel. When the coated and uncoated alginate beads were incubated in citrate (2.5–5% *w*/*v*), the uncoated alginate capsule was completely dissolved. In contrast, PLL-coated alginate beads, as well as the dual layer having the interpenetrating network, persisted in a 5% citrate solution for over 14 h at room temperature (Figure 7 and Figure S4). This shows the protective effect of the PLL layers on the materials encapsulated within the alginate beads. Thus, regardless of any loss of cross-links from the alginate core, the poly-lysine layers will ensure that the microcapsules preserve their structural integrity, similar to the report of Gugerli et al. [28]. Additionally, the dried beads regained their sphericity upon rehydration.

*Pseudomonas aeruginosa* contains two transcription regulators (LasR and RhlR) that, when complexed with their specific autoinducers (3-oxo-C_12_-HSl and C_4_-HSL, respectively), activate the transcription of different virulence-associated traits and enzymes involved in rhamnolipid biosynthesis [40]. In this study, mutant strains of *Pseudomonas aeruginosa* were utilized. These strains have a knockout on the gene expressing 3-oxo-C_12_-HSL and C_4_-HSL autoinducers, necessitating the exogenous addition of the autoinducer for the activation of the transcription of the promoter-controlled bacterial genes fused to the reporter (luciferase) gene (Figure S1). Consequently, the expression of the reporter genes in the mutant bioreporter can be correlated with the amount of exogenously added autoinducers in the media, forming the basis for detecting QS molecules (autoinducers) using bioreporter bacterial strains.

Two mutant strains of *P. aeruginosa* (LasR and RhlR) were genetically engineered to express bioluminescence in response to specific quorum-sensing (QS) molecules. These strains were individually encapsulated within alginate-PLL beads following the procedure shown in Figure 2. LasR and RhlR can quantitatively respond to 3-oxo-C_12_-HSL and C_4_-HSL, respectively. Increased bioluminescence intensity was observed with increasing concentrations of the AHL compounds used. Upon preparation, the bioreporter-loaded microcapsules were incubated in a culture media overnight (20 h) at 30 °C and 150 rpm. Subsequently, viability testing was performed, and confocal imaging confirmed the viability of the encapsulated bioreporter. The bioreporter-loaded beads were subjected to various concentrations (including the absence) of autoinducers, and the resulting bioluminescence response was recorded. The obtained results provide evidence that the bacteria remain viable and exhibit their genetically programmed response, thus retaining the sensing functionality.

The plasmids used in this study were constructed by taking advantage of the RhlI/RhlR and LasI/LasR quorum-sensing systems of the Gram-negative bacterium *P. aeruginosa*. C_4_-HSL and 3-oxo-C_12_-HSL are known to be the respective cognate activator molecules, i.e., the natural ligands that *P. aeruginosa* synthesize, sense, and respond to [41]. In this study, we observed increased bioluminescence intensity with increasing concentrations of C_4_-HSL and 3-oxo-C_12_-HSL. The RhlR-based bioreporter beads demonstrated a dynamic range and limit of detection of 10 µM–0.1 nM and 50 pM, respectively, for C_4_-HSL. In contrast, the LasR-based bioreporter showed a dynamic range and a detection limit of 5 µM–0.2 nM and 0.1 nM, respectively, for 3-oxo-C_12_-HSL. A calibration curve was generated with either C_4_-HSL or 3-oxo-C_12_-HSL at known concentrations, which can be used to calculate the concentration of C_4_-HSL or 3oxo-C_12_-HSL activity in each sample. The molecule C_4_-HSL, at a concentration of 10 µM, induced the maximum response in the RhlR system carrying the plasmid pKD-rhlA. Meanwhile, 2 µM of the 3-oxo-C_12_-HSL molecule exhibited a maximum response in the bioreporter containing the plasmid pKD201 lasl. These findings align with previous studies, indicating that long-chain AHLs induce the LasR regulatory system to a greater extent, whereas short-chain AHLs efficiently activate the RhlR regulatory system [42]. Additionally, the limit of detection (LOD) was defined as the lowest concentration of AHL capable of inducing a 20% increase in luminescence by the biosensor without any added molecule.

The wide range of response and the high sensitivity of these bioreporters suggest that the bacteria strain in this study could be used for the direct determination of the 3-oxo-C_12_-HSL and C4-HSL levels in *P. aeruginosa* laboratory cultures and clinical samples where *P. aeruginosa* is implicated. According to previous reports, the highest concentration of AHLs in laboratory cultures and clinical samples (cystic fibrosis sputa) is ≤28 μM and ≤21 nM, respectively [8,43].

After assay optimization, our whole-cell sensing systems were employed to detect the presence of the AHLs in samples collected from cell-free biofilm and planktonic bacteria cultures. We also evaluated our whole-cell sensing systems for their ability to detect the presence of quorum-sensing inhibitors (QSI) by measuring the residual QS activity in the presence of autoinducers and QSI. To this end, we tested our system’s response to furanone C-30 (FC30), a well-known QSI molecule that interferes with RhlR and LasR QS signaling [44]. The system exhibited a similar inhibition response to FC30 when tested in the presence of either synthetic autoinducers (Figure 14) or a cell-free culture of the PAO1 strain of *P. aeruginosa* (Figure S15). Furthermore, the system maintained its sensing ability when stored at 4 °C for 60 days (Figure 15) and when stored for over 85 days) and frozen at −80 °C. Lyophilization resulted in a significant loss of bioluminescence response, but simple air drying of the bioreporter did not affect its sensing capability. The biosensor strains did not require special treatment to revive them after long storage; they existed as ready-to-deploy microcapsules. Overall, the bioreporter encapsulated in alginate-PLL microbeads has the potential to be a sensitive, easy-to-prepare, and easy-to-deploy analytical unit suitable for various biotechnological applications in both developed and low-income countries.

## 5. Conclusions

We developed hydrogel-immobilized bioreporters as on-demand, whole-cell biosensing systems for the sensitive, selective, and rapid detection of AHLs in spiked samples and laboratory cultures. The method is simple, sensitive, reproducible, and amenable for point-of-need deployment without extensive sample preparation. The RhlR-based bioreporter beads demonstrate a dynamic range and limit of detection of 10 µM–0.1 nM and 50 pM, respectively, for C_4_-HSL, while the LasR-based bioreporter showed a dynamic range and a limit of detection of 5 µM–0.2 M and 0.1 nM, respectively, for 3-oxo-C_12_-HSL.

The immobilized bioreporters can be stored stably and refrigerated as ready-to-use microbeads for over 60 days. Furthermore, the developed method is cost-effective, robust, and adaptable for screening quorum-sensing (QS) inhibitors. We believe this novel biosensing system could be utilized for diagnosis and environmental monitoring. Additionally, our systems could serve as a valuable tool for screening novel QSI-based drugs active against *Pseudomonas aeruginosa* infection and a variety of diseases that involve QS in their pathogenesis.

## Data Availability

The original results presented in this study are included in the article and the supplementary materials; further inquiries in terms of data can be found in our records in footprints, and authorization for its access may be granted by the corresponding author (rsmarks@bgu.ac.il).

## Supplementary Materials

The following supporting information can be downloaded at: https://www.mdpi.com/article/10.3390/bios14080383/s1. Figure S1: The plasmids used in this study; Figure S2: The structures of QS molecules used in this study; Figure S3: Bead sizes are a function of air pressure; Figure S4: Alginate-PLL IPN showed enhanced stability chelators; Figure S5: The ATR-FTIR spectra of calcium alginate, sodium alginate, and calcium alginate-poly-lysine; Figure S6: XPS atomic peak composition of the lyophilized alginate-poly-lysine beads; Figure S7: The micrograph of live/dead, stained bioreporter beads; Figure S8: Bioluminescent measurement normalized by cell density; Figure S9: The dose-dependent response of the LasR bioreporter; Figure S10: Different layers of the microbead performances; Figure S11: Storage for 48 h in a refrigerator and freezer; Figure S12: Flow-cell biofilm experiment; Protocol S1: The detection of AHLs in the biofilm effluent obtained in the flow-cell experiment; Protocol S2: Biofilm visualization; Figure S13: Typical visualization of the biofilm after 24, 48, and 72 h; Figure S14: The bioluminescent response of the bioreporters to biofilm effluents; Figure S15: Inhibitory effect of furanone C-30 (FC30) on the activity of RhlR; Figure S16: At higher concentrations of F-C30, the bioluminescence of the RhlR reporter was almost quenched; Figure S17: Activity of the lyophilized LasR beads; Figure S18: The intermachine differences; and Figure S19: Optical-fiber, whole-cell biosensors. Reference [29] is cited in the supplementary materials.
