# Supplementary material for: Microbead-Encapsulated Luminescent Bioreporter Screening of P. aeruginosa via Its Secreted Quorum-Sensing Molecules
Source: Biosensors (Basel). 2024 Aug 8;14(8):383. doi: 10.3390/bios14080383 (PMC11352650; doi:10.3390/bios14080383)
Supplement: Supplementary file 1 [file biosensors-14-00383-s001.zip › biosensors-3081129-supplementary.pdf]

Supplementary Materials

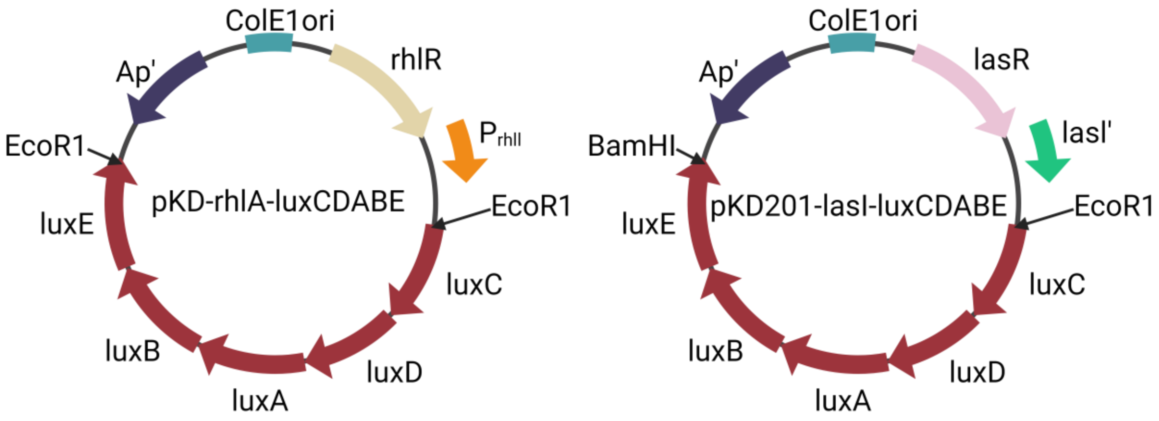

Figure S1. The plasmids used in the study.

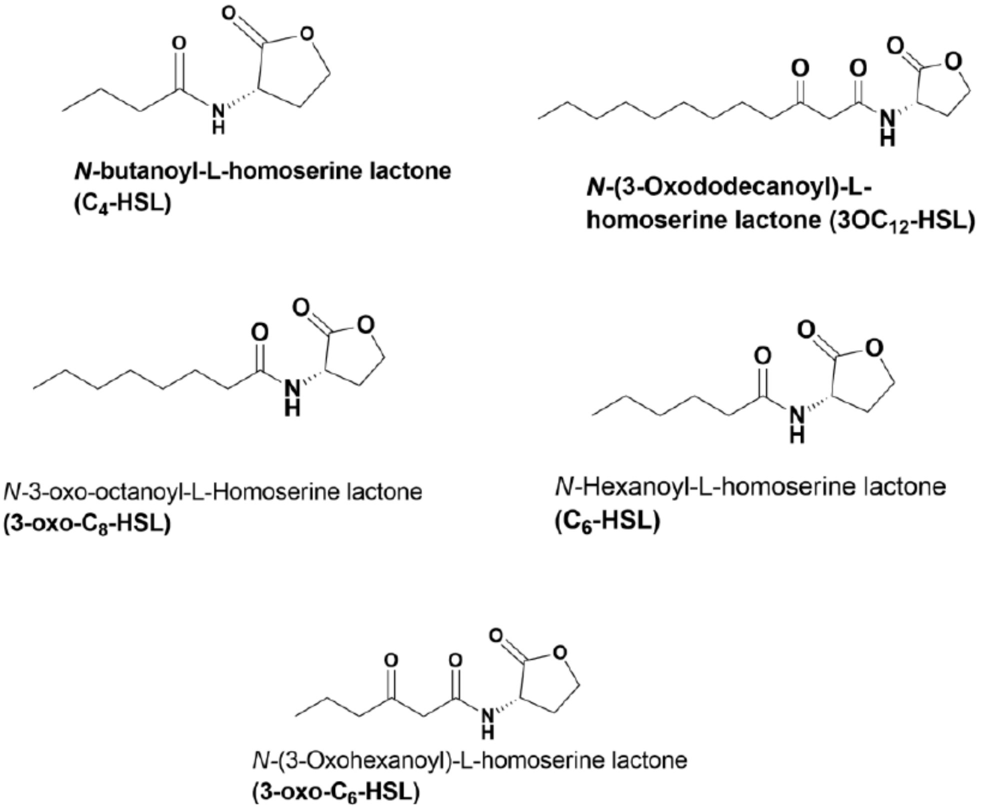

Figure S2. The structures of QS molecules used in the study. C4-HSL and 3-oxo-C12-HSL are well-characterized *P. aeruginosa* autoinducers of RhlR and LasR quorum-sensing signaling, respectively.

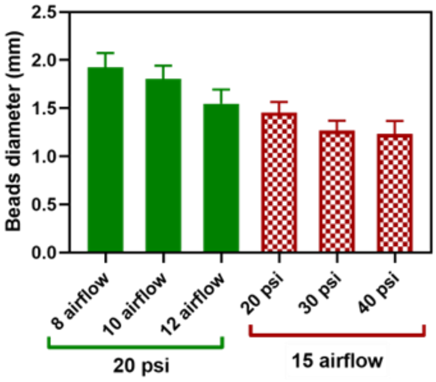

Figure S3. Bead sizes are a function of air pressure.

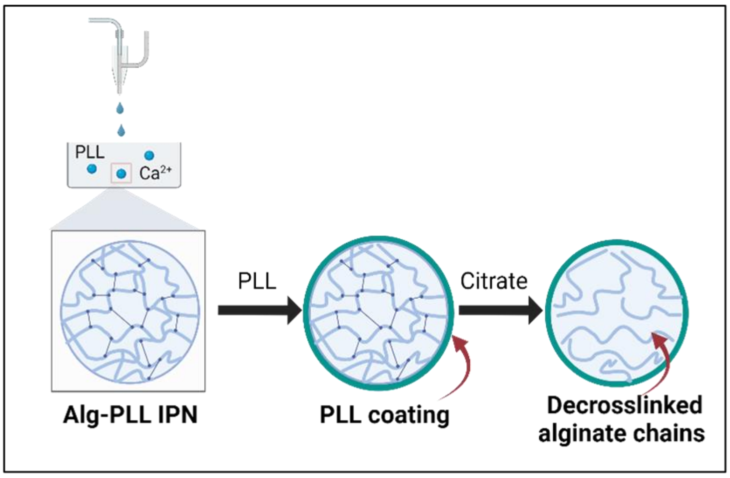

Figure S4. The alginate-PLL IPN showed enhanced stability chelators.

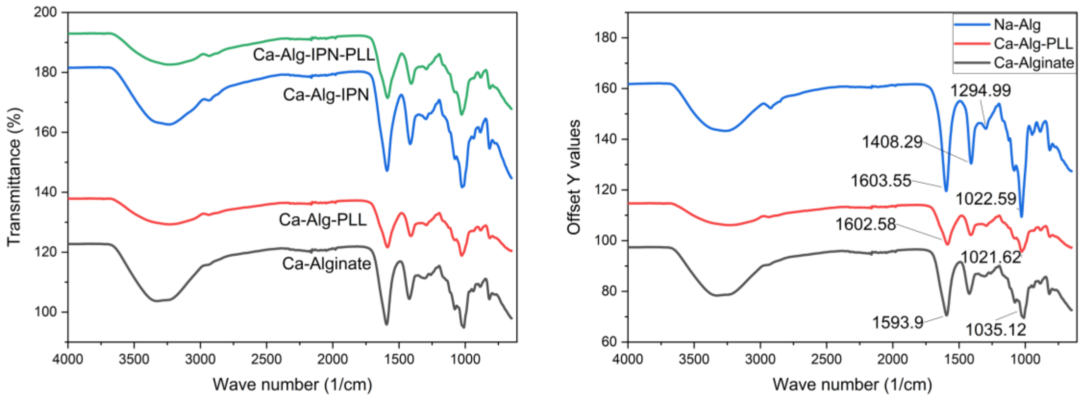

Figure S5. The ATR-FTIR Spectra of calcium alginate, sodium alginate, and calcium alginate-poly-lysine.

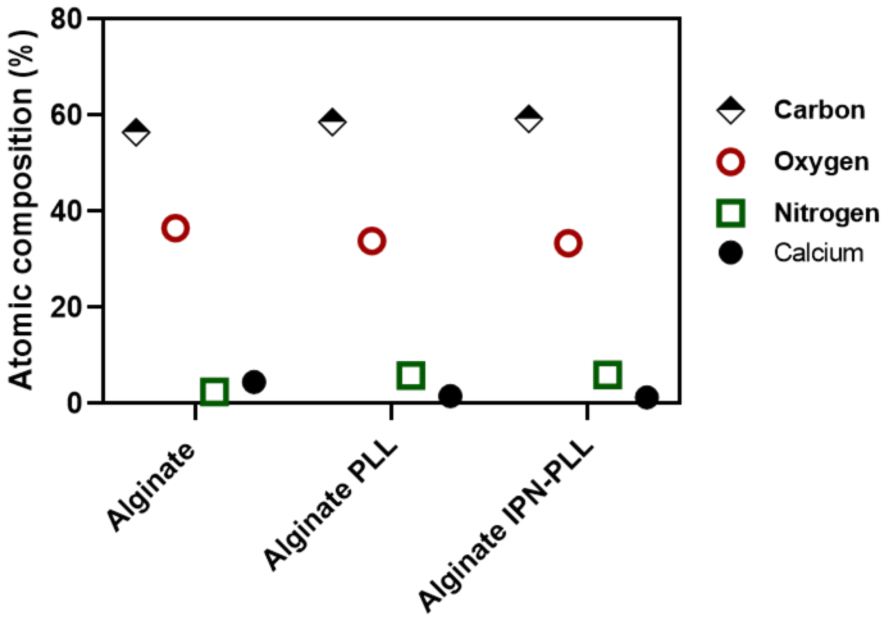

Figure S6. The XPS atomic peak composition of the lyophilized alginate-poly-lysine beads.

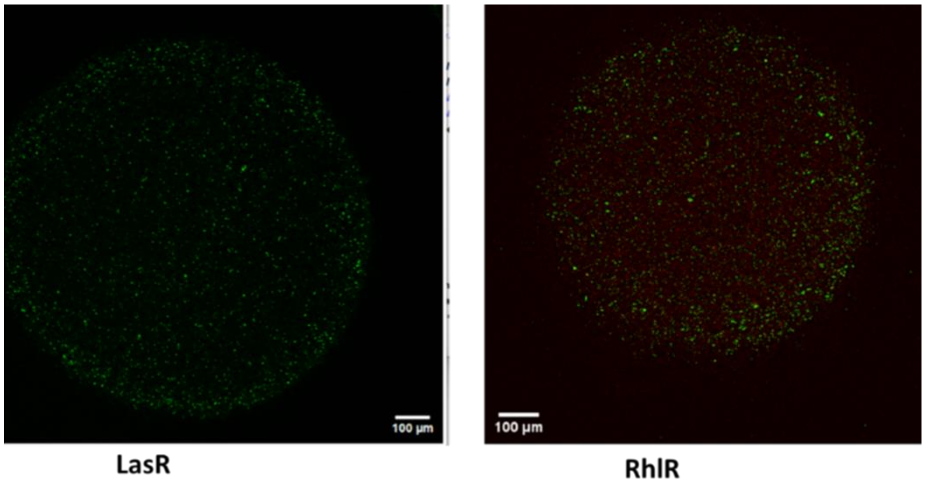

Figure S7. The micrograph of live/dead, stained bioreporter beads. Microcapsules containing LasR and RhlR strains were placed in a sterile 24-well plate containing LB broth and were mildly shaken for about 25 hours in an orbital shaker in a hot room maintained at 30 °C. For live/dead staining, the capsules were washed with a saline solution (0.9% w/v NaCl) three times. After washing, the capsules were incubated at room temperature for 15 min in a saline solution containing propidium iodide (dead) and SYTO™ 9 (live), which were then stain-washed with saline and transferred to the ibid 12-well plate for confocal microscope imaging.

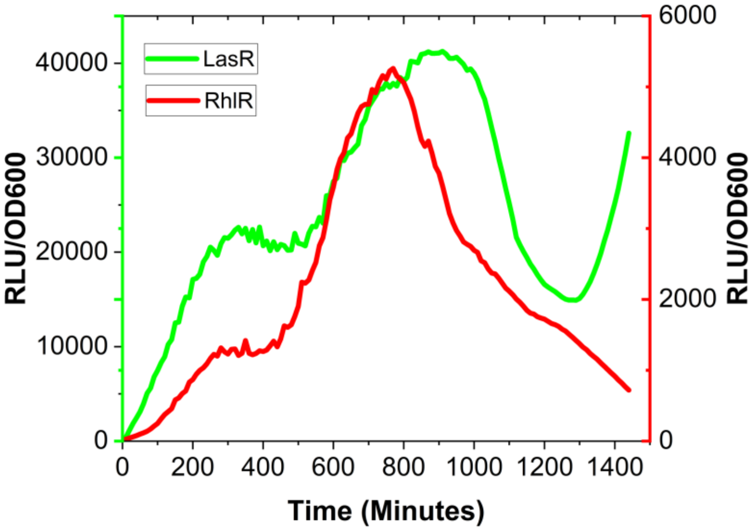

Figure S8. The bioluminescent measurement normalized by the cell density. The spectra of each reporter strain were normalized by the cell density (OD600) at any given time point. 5 M, 2 M, 0.2 M, 5 nM, 2 nM, 0.2 nM, 0, 50, 100, and 150 LasR calibration by IF concentration of the 3-oxo-C12-HSL induction factor.

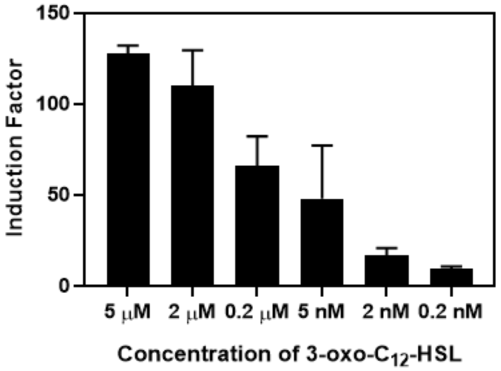

Figure S9. The dose-dependent response of the LasR bioreporter carried out as described in Section 2.5.2 in the main text..

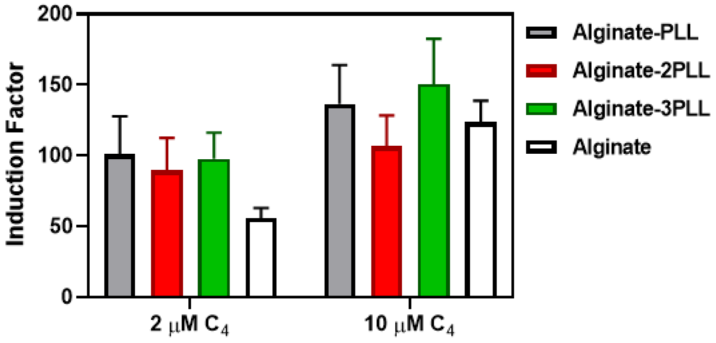

Figure S10. Different layers of the microbead performances. The different poly-lysine coating layers were prepared and tested for bioluminescent activity. For the sake of structural strength, all experiments were carried out using three layers of poly-lysine coating, as shown in the main text.

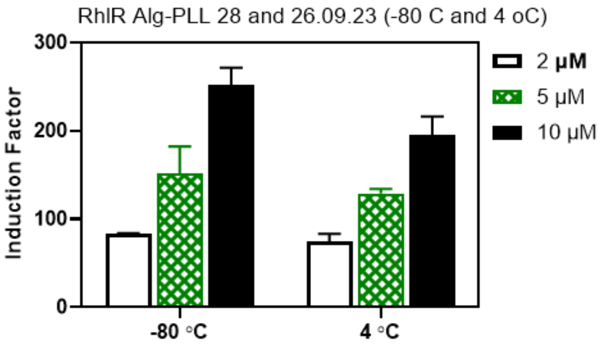

Figure S11. Storage for 48 h in a refrigerator and freezer. The activities of the reporter bacteria stored at 4 °C and -80 °C (48 h) were determined and compared (Figure 6).

Protocol S1*. The detection of AHLs in the biofilm effluent obtained in the flow-cell experiment.*
The biofilm experiment was carried out under flow conditions using a standard method [1], as shown in the following scheme (Figure S2). Briefly, to initiate biofilm growth, overnight cultures of *P. aeruginosa*, *P. baumanni*, or *E. coli* were diluted with a saline solution (0.9% NaCl) to an OD600 of about 0.1 and 250 μL of the diluted cultures, which were then injected into separate static Ibidi flow-cells (μ-slide sizes of 17 × 3.8 × 0.4 mm). After 1 h of incubation under static conditions to allow bacterial adhesion, the flow was introduced with a constant flow rate of 3 mL of h−1 (Masterflex L/S peristaltic pump, Cole-Parmer, Vernon Hills, IL, USA) of 10% tryptic soy broth (TSB) supplemented with 0.1% glucose. The flow was allowed to continue undisturbed for 12, 24, 48, and 72 h, and the flow through (effluent) at the determined time was collected, filtered using a 0.22 μm-membrane filter, and stored at -80 °C until required. The flow through was used as the source of AHLs for the biosensor testing. The resulting biofilms were macroscopically visualized using the CLSM and 3D processed by IMARIS software, as shown in the next sections.

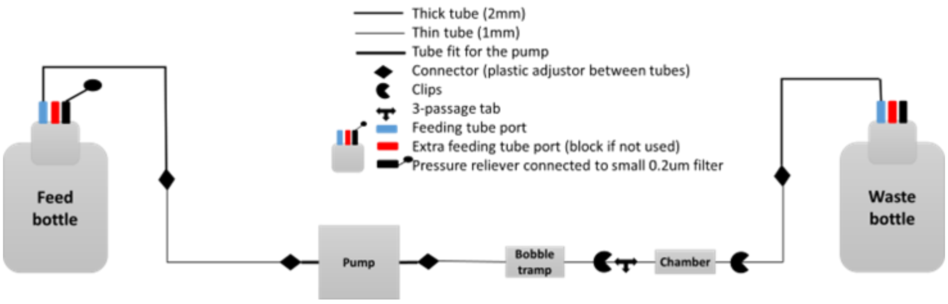

Figure S12. Flow-cell biofilm experiment.

Protocol S2*. Biofilm Visualization*
The biofilm's thickness and architecture were acquired using a confocal laser scanning microscope (CLSM) manufactured by Olympus in Tokyo, Japan. The microscope was equipped with a 60 × 1.35NA lens. To detect the green-live SYTO 9 dye, an excitation wavelength of 488 nm was used, and the emission wavelengths of 515 nm were collected. Similarly, for detecting the red-dead PI stain, excitation was conducted at 530 nm, and the emission was recorded at 617 nm. Using the Easy 3D function of the IMARIS software, three-dimensional projections of the biofilm structures were reconstructed while incorporating the quantitative structural parameters of the biofilms recorded as biovolume.

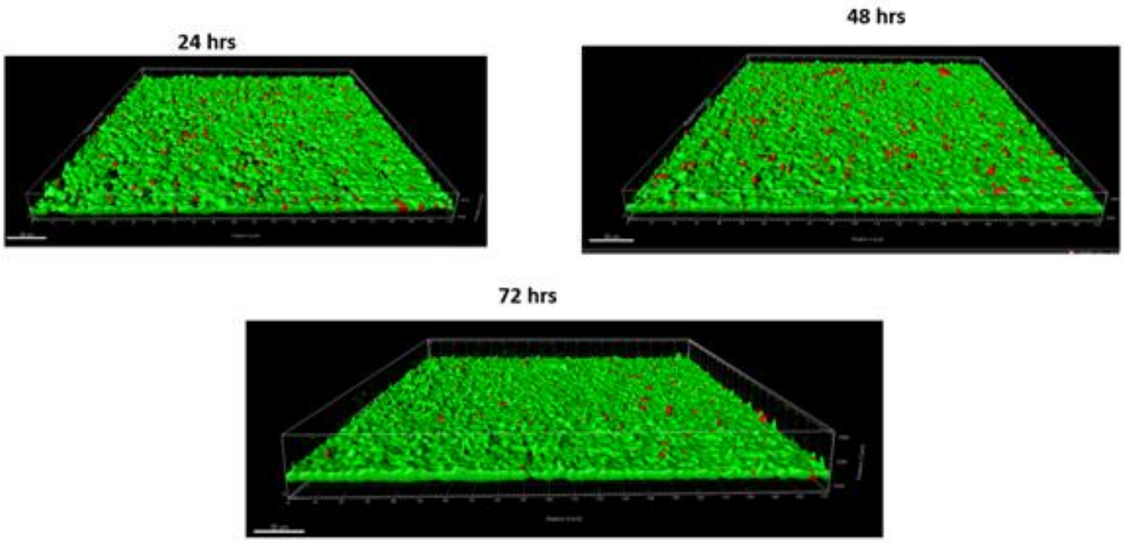

Figure S13. Typical visualization of the biofilm after 24, 48, and 72 hrs.

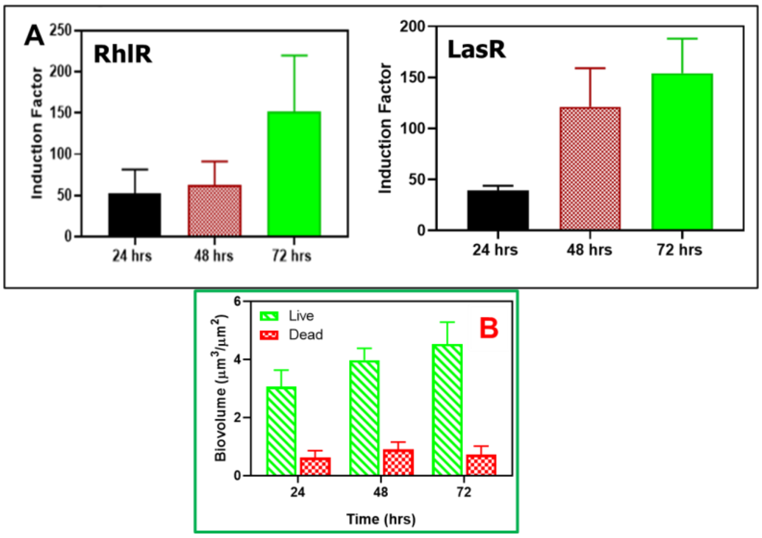

Figure S14. The bioluminescent response of the bioreporters to biofilm effluents (Panel A), and the biovolume determined by confocal microscopy (Panel B).

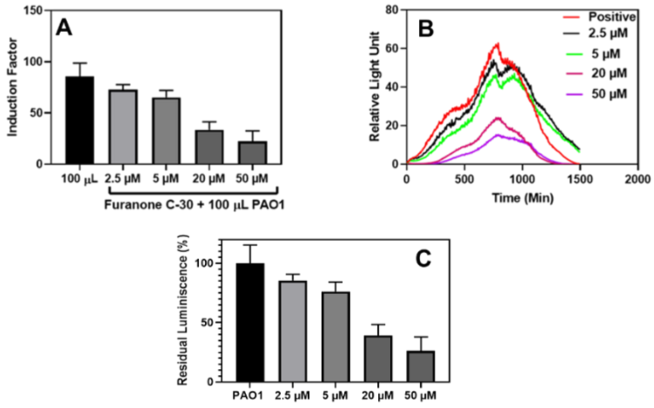

Figure S15. The inhibitory effect of furanone C-30 (FC30) on the activity of RhlR in the presence of the cell-free supernatant of *P. aeruginosa* PAO1 (100 μL) expressed as the induction factor (A), spectrum (B), and percentage (C).

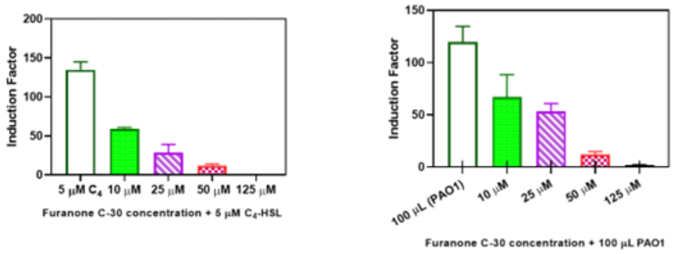

Figure S16. At higher concentrations of F-C30, the bioluminescence of the RhlR reporter was almost quenched.

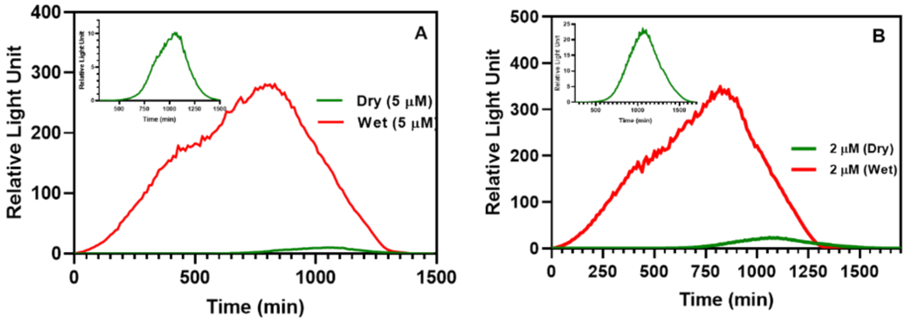

Figure S17: Activity of the lyophilized LasR beads. (A) After 4 hours of lyophilization and (B) after 2 hours of lyophilization. The green line represents the luminescence obtained from the lyophilized samples, while the red line shows the activity of the fresh beads stored in the refrigerator prior to the bioluminescent assay. The spectra lines show the average values of three beads per well in four replicate experiments

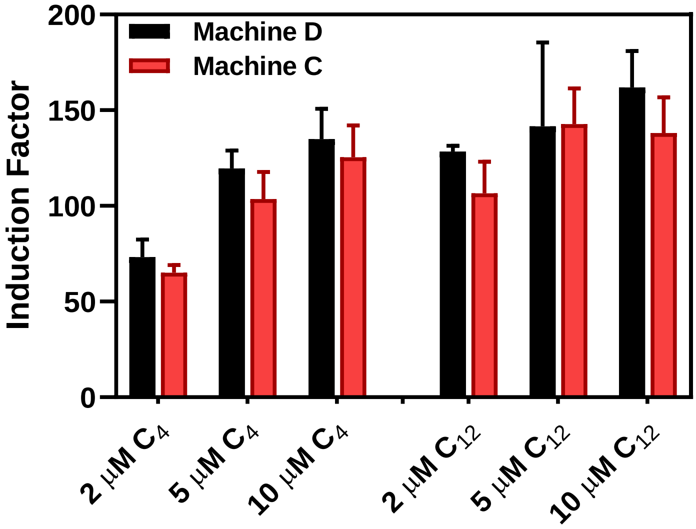

Figure S18. The intermachine differences. After 38 days of storage at 4 °C, two identical plates were prepared consisting of the encapsulated RhlR strain incubated with different concentrations of autoinducers. The bioluminescent responses of each plate were recorded simultaneously using two different luminometers. The intermachine differences between each concentration tested were not significantly different ($p < 0.05$).

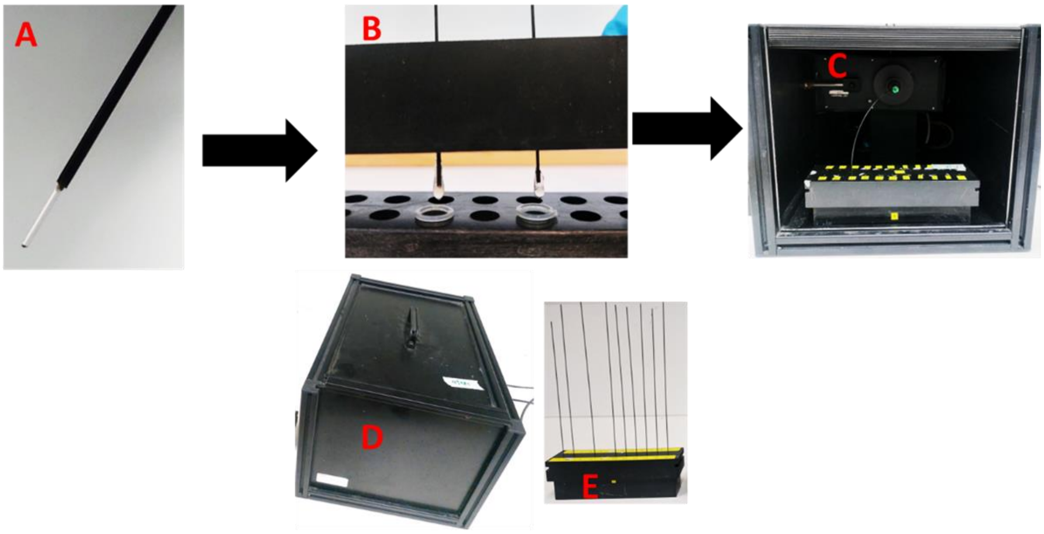

Figure S19. Optical-fiber, whole-cell biosensors. (A) The stripped optical fiber. (B) The bioreporter immobilized unto the striped optical fiber. (C) The bioreporter optical fiber inserted into the analytes and connected to the photon-counting unit (PMT) via fiber holder. (D) Picture of the closed biosensor box. (E) Picture of the optical fibers inserted into the sample holder.

1. Golberg, K.; Markus, V.; Kagan, B.-e.; Barzanizan, S.; Yaniv, K.; Teralı, K.; Kramarsky-Winter, E.; Marks, R.S.; Kushmaro, A.J.P. Anti-Virulence Activity of 3, 3′ -Diindolylmethane (DIM): A Bioactive Cruciferous Phytochemical with Accelerated Wound Healing Benefits. **2022**, *14*, 967.
